# Supplementary material for: Individual and interpersonal factors influencing child marriage: A qualitative content analysis study
Source: PLoS One. 2024 Nov 19;19(11):e0313933. doi: 10.1371/journal.pone.0313933 (PMC11575807; doi:10.1371/journal.pone.0313933)
Supplement: S2 Table — (DOCX) [file pone.0313933.s002.docx]

# **Table S2: Demographic characteristics of women’s who married under the age of 15**

| **Participants** | **Marriage Age** | **Current Age** | **Husband's Age****(Time Of Marriage)** | **Residence** |
| --- | --- | --- | --- | --- |
| 1 | 14 | 15 | 21 | Rural |
| 2 | 14 | 16 | 18 | Urban |
| 3 | 14 | 15 | 17 | Urban |
| 4 | 14 | 21 | 21 | Rural |
| 5 | 14 | 22 | 21 | Urban |
| 6 | 14 | 22 | 20 | Urban |
| 7 | 13 | 20 | 17 | Rural |
| 8 | 14 | 27 | 24 | Rural |
| 9 | 14 | 29 | 18 | Rural |
| 10 | 13 | 20 | 25 | Urban |
| 11 | 14 | 24 | 24 | Rural |
| 12 | 13 | 20 | 24 | Urban |
| 13 | 14 | 29 | 24 | Urban |
| 14 | 14 | 28 | 24 | Rural |
| 15 | 14 | 19 | 20 | Urban |

# **Note: The diversity of participants in terms of current age is shown in the table**
